# Butyric Acid Protects Against Renal Ischemia–Reperfusion Injury by Adjusting the Treg/Th17 Balance *via* HO-1/p-STAT3 Signaling

**DOI:** 10.3389/fcell.2021.733308

**Published:** 2021-11-02

**Authors:** Zhen Chen, Miaomiao Wang, Shikun Yang, Jian Shi, Tianhao Ji, Wei Ding, Lianghua Jiang, Zhiwen Fan, Jing Chen, Yunjie Lu

**Affiliations:** ^1^The Third Affiliated Hospital of Soochow University, Changzhou, China; ^2^Key Laboratory of Liver Transplantation, Hepatobiliary/Liver Transplantation Center, The First Affiliated Hospital of Nanjing Medical University, Chinese Academy of Medical Sciences, Nanjing, China; ^3^Wujin Hospital Affiliated With Jiangsu University, Changzhou, China; ^4^The First People’s Hospital of Kunshan, Kunshan, China; ^5^Department of Pathology, Affiliated Nanjing Drum Tower Hospital of Nanjing University School of Medicine, Nanjing, China

**Keywords:** butyric acid, renal ischemia–reperfusion injury, Treg, Th17, HO-1, STAT3

## Abstract

Immune regulation plays a vital role in ischemia–reperfusion injury (IRI). Butyric acid (BA) has immunomodulatory effects in many diseases, but its immunomodulatory effects during renal IRI are still unclear. Our research shows that BA protected against IRI and significantly improved renal IRI *in vivo*. *In vitro* studies showed that BA inhibits Th17 cell differentiation and induces Treg cell differentiation. Mechanism studies have shown that heme oxygenase 1 (HO-1)/STAT3 signaling pathway was involved in the inhibitory effect of BA on Th17 cell differentiation. HO-1 inhibitors can significantly rescue the BA-mediated inhibition of Th17 cell differentiation. We confirmed that BA promotes the differentiation of Th17 cells into Treg cells by regulating the pathway and reduces renal IRI.

## Introduction

The kidney is a richly perfused organ and is very sensitive to ischemia–reperfusion injury (IRI). Complex physiological processes are involved in renal IRI, such as inflammatory T cells and cytokines, which play an essential role in the process of renal injury (Sakai et al., 2019). Regulatory T cells (Tregs), formerly known as suppressor T cells, are a subpopulation of T lymphocytes that modulate the immune system, maintain tolerance to self-antigens, and abrogate autoimmune disease (Takahashi et al., 1998; Barbi et al., 2014). Tregs express CD4 and CD25 on their surface, and the transcription factor forkhead box p3 (Foxp3) is the critical marker for Treg function (Lu et al., 2017). Previous studies have indicated that a moderate population of Treg is necessary for immune homeostasis (Shevyrev and Tereshchenko, 2019). Although the exact etiology and pathogenesis of renal IRI are not well-defined, current research showed that Tregs protected the kidney against IRI, which were involved in renal tubular epithelial cells, and the imbalance of Th17/Treg is critical in renal IRI processes (Suzuki et al., 2019; Dellepiane et al., 2020). Treg could be the potential treatment in IRI (Brunstein et al., 2016; Lu et al., 2016; Yamamoto et al., 2020). Thus, maintaining Treg stability and function in IRI patients has become a key research direction in immunology.

The microbiome of the intestine and its products could modulate inflammatory reactions by taking part in controlling the activity of Tregs, and the responses can be observed in other organs, such as the central nervous and airway systems (Ochoa-Reparaz et al., 2009; Trompette et al., 2014). The butyric acid (BA) is the product of indigestible foods fermented by intestinal microbial, which has a small molecular structure and belongs to the short-chain fatty acid (SCFA) family of microbiome products (Maslowski et al., 2009; Furusawa et al., 2013). As is shown in recent works, BA is actively involved in several pathological processes, including autoimmunity, cancer, and neurological disorders (Arpaia et al., 2013; Singh et al., 2014; Stilling et al., 2016). Moreover, according to several recent studies, the BA shows a regulatory effect of Treg induction *in vivo* and *in vitro* (Arpaia et al., 2013; Smith et al., 2013). Therefore, BA may be a potential negative immunoregulative agent that can be used to treat multiple diseases. However, whether BA can inhibit renal IRI has not been explored. Moreover, it is still unclear that how BA mediates immunomodulation.

In the present study, we attempted to use BA to definite the role of BA in renal IRI and explore the possible immunomodulatory mechanism. Our results strongly suggest that BA directly inhibits Th17 cell differentiation and induces Treg cell differentiation to protect renal IRI.

## Materials and Methods

### Cell Acquisition and Culture

Peripheral blood was obtained from volunteers from The Department of Urology of the Third Affiliated Hospital of Soochow University. Human naïve CD4^+^ T cells (CD4^+^CD25^–^CD45RA^+^) were isolated and purified from peripheral blood mononuclear cells (PBMCs) using Ficoll-Hypaque separation (Amersham Biosciences) and the two-step magnetic bead separation method of an autoMACS Pro Separator (Miltenyi Biotechnology Company, Germany).

Naïve CD4^+^ T cells were induced to CD4^+^ iTregs with TGF-β (5 ng/ml) (Bio-techne, Abingdon, OX, United Kingdom), recombinant IL-2 (100 IU/ml) (Chiron, Emeryville, CA, United States), and antihuman CD3/CD28 beads (ratio of cells to beads = 1:5) (Life Technology, Carlsbad, CA, United States) in X-Vivo-15 medium (BioWhittaker, Walkersville, MD, United States) containing 10% fetal bovine serum (Cereal Biomedicine) for 6 days. The BA (100 μmol/L) used in cell culture was prepared by dissolving BA sodium powder (Sigma-Aldrich, 303410) in dimethyl sulfoxide (DMSO).

The PC61 α-CD25 antibody (Biolegend Cat#102007) was generated from hybridoma cells (ATCC) in serum-free media (Gibco).

### Experimental Animals and Renal Ischemia–Reperfusion Injury Model

We constructed the renal IRI model according to the conventional method (Hosszu et al., 2017). Male C57BL/6 mice (20–25 g) 7–8 weeks of age were purchased from Kawensi Biotech (Changzhou, China). We established the mouse model of renal IRI by clamping the bilateral renal pedicles for 45 min. A warming pad was used to keep body temperature around 37°C during the surgery. After 45 min of ischemia, the needle was withdrawn to allow reperfusion for 1 day. Similar surgical procedures except clamping of the renal pedicles were applied to the sham-operated group. The animal experiments were approved by the Animal Management and Use Committee of Soochow University.

### Interventions of Animal Model

Mice were given 100 mM BA in their drinking water. The renal IRI model was established after 6 days. Mice were sacrificed on post-operative day 1. Blood samples were collected from the tail vein to examine creatinine (Cr) and blood urea nitrogen (BUN) levels. Spleens and kidneys from the rats were obtained and fixed in 10% neutral formalin for 48–72 h. The specimens were dehydrated by a graded ethanol series and embedded in paraffin. Kidney tissue sections were subjected to hematoxylin-eosin (HE) staining, and the kidney tubule injury was assessed using the Paller score. Ten non-overlapping visual fields (200× microscope) were randomly selected from each rat and observed under a light microscope. For each field of view, 10 kidney tubules were randomly chosen for quantification. A total of 100 renal tubules were scored, and the higher the score was, the more serious the tubular injury was Paller et al. (1984).

### Quantitative Real-Time Polymerase Chain Reaction

We tested the expression of IL-17A, IFN-γ, and IL-10 mRNA in tubules of mice. Total RNA extracted from tissue and cell lysates was further purified using RNeasy Mini Kit (Qiagen, Valencia, CA, United States). We obtained cDNA using Omniscript RT kit (Qiagen, Netherlands). Relative quantification of mRNA expression levels was performed using Absolute QPCR SYBR Green ROX Mix (Thermo Fisher Scientific Inc., Waltham, MA, United States). The relative mRNA levels were assessed (in triplicate) based upon normalization using a reference gene encoding β-actin (Actb).

### Western Blotting Assay

The total protein was extracted by radioimmunoassay lysate with 1% protease inhibitor on ice. The protein concentration was measured by BCA protein analysis kit (Thermo Fisher Scientific, United States). The proteins were separated by sodium dodecyl sulfate polyacrylamide gel electrophoresis, and transferred to PVDF membrane. After blocking with 5% fresh milk for 1 h, the membrane was cultured overnight with specific primary antibody at 4°C and then cultured with corresponding secondary antibody for 30 min. Finally, the strips on the film were detected by ECL detection system (Thermo Fisher Scientific, United States) and quantified by Quantity One software (v4.3 in the United States).

### Enzyme-Linked Immunosorbent Assay

Enzyme linked immunosorbent assay (ELISA) was performed to detect quantitatively inflammatory cytokine. Blood from the inferior vena cava was left for 20 min and then collected into tubes and centrifuged (2,000 rpm, 15 min, 4°C). Then 3 ml supernatants of kidney homogenates were harvested and stored at −20°C. IL-17A, IFN-γ, and IL-10 were measured by ELISA (R&D Systems) according to the product instruction.

### Flow Cytometry

(1) Flow cytometry was used to measure the percentage of Th17 cells. A 250-μl whole blood sample was mixed with 250 μl RPMI-1640 medium without fetal bovine serum to a volume of 500 μl. Individual control and experimental tubes were set up. Then 250 μl diluent, 1 μl PMA (0.1 mg/ml), 5 μl ionomycin (1 mg/ml), and 1 μl monensin (50 mg/ml) were added to each tube, incubated at 37°C for 12 h in a 5% CO_2_ incubator. The mixture was centrifuged again and the supernatant was discarded. Then red cells were lysed by adding a 1-ml lysing buffer (FACS lysing solution; Bio Legend Co.). Cells were incubated for 5 min at room temperature. The mixture was centrifuged again and the supernatant again discarded. The cells were resuspended in PBS and transferred into a 1.5-ml Eppendorf tube. The cells were centrifuged at 2,500 rpm for 5 min, and the supernatant was discarded. The wells were washed twice with PBS, added with 1 μl anti-mouse CD3FITC (0.5 mg/ml) and 1 μl anti-rabbit CD8aPE (0.2 mg/ml), and kept at room temperature away from light for 30 min. The wells were washed once with PBS and the supernatant was discarded, then added with 0.5 ml fixatives and protected from light for 20 min for incubation at room temperature. The cells were washed once with 1 ml PBS, centrifuged at 1,000 rpm for 5 min, and the supernatant was discarded. The cells were added with 1 ml permeabilization wash buffer and protected from light for 20 min for incubation at room temperature, centrifuged at 1,000 rpm for 5 min, and the supernatant was discarded. After washing with PBS, 1 μl anti-rabbit IL-17AAPC (0.2 mg/ml) was added into experimental tubes and 1 μl APC-lgG1 was added into control tubes. The cells were protected from light for 30 min for incubation at 4°C, washed once with PBS, centrifuged at 1,000 rpm for 5 min, and the supernatant was discarded. The cells were resuspended in 0.5 ml PBS and analyzed for CD3^+^CD8^–^IL-17^+^ expression by flow cytometry.

(2) Flow cytometry was used to measure the percentage of Treg cells. Individual control and experimental tubes were set up. Then 100 μl whole blood, 1 μl anti-mouse CD4ECD, and 1 μl anti-mouse CD25PE were added to each tube, kept at room temperature away from light for 30 min, centrifuged at 1,000 rpm for 5 min, and the supernatant was discarded. The cells were washed once with 1 ml PBS, centrifuged at 1,000 rpm for 5 min, and the supernatant was discarded. Then red cells were lysed by adding a 1-ml lysing buffer (FACS lysing solution; Bio Legend Co.). Cells were incubated for 5 min at room temperature. The cells were added with 0.5 ml Foxp3 fixatives and protected from light for 20 min for incubation at room temperature. The cells were washed once with 1 ml PBS, centrifuged at 1,000 rpm for 5 min, and the supernatant was discarded. The cells were added with 1 ml permeabilization wash buffer and protected from light for 20 min for incubation at room temperature, centrifuged at 1,000 rpm for 5 min, and the supernatant was discarded. After washing with PBS, 1 μl anti-mouse Foxp3Percpcy5 was added into experimental tubes and 1 μl Percpcy-lgG2 was added into control tubes. The cells were protected from light for 30 min for incubation at room temperature, washed once with PBS, centrifuged at 1,000 rpm for 5 min, and the supernatant was discarded. The cells were resuspended in 0.5 ml PBS and analyzed for CD4^+^CD25^+^Foxp3^+^ expression by flow cytometry.

### Statistical Analysis

Stata software (version 11.0) was used to perform the analysis. Data were expressed as mean ± SD; the differences between groups were analyzed by either the paired *t*-test or ANOVA test (both one-way ANOVA test and two-way ANOVA test). In addition, we use Bonferroni or LSD as a *post hoc* test. *P*-values < 0.05 (two-tailed) were considered statistically significant.

## Results

### Butyric Acid Treatment Ameliorated Renal Ischemia–Reperfusion Injury

**FIGURE 1:**
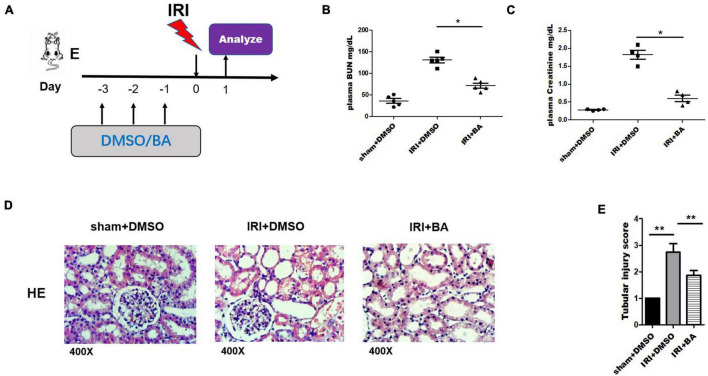
BA treatment ameliorated renal IRI. **(A)** Mice were orally administered BA and DMSO 3 days before surgery. **(B,C)** BUN and Cr of mice on the first day after surgery were tested. **(D)** HE staining of kidneys in the BA treatment group showed alleviate renal tubular damage, renal tubular epithelial cells atrophy, degeneration, necrosis, and extensive lesion range than control group. **(E)** BA treatment significantly reduced the tubular injury Paller score in IRI mice. Sample size = 3 in each group. Data were presented as mean ± standard deviation. N.S. *P* > 0.05, *P < 0.05, **P < 0.01.

Mice were orally administered BA and DMSO 3 days before surgery as indicated in Figure 1A. We tested the BUN and Cr of mice on the first day after surgery and obtained kidney tissue specimens. BA significantly reduced the increase of BUN and Cr in mice caused by IRI, suggesting the protective effect of BA on the renal function of IRI in mice (Figures 1B,C). HE staining of kidneys in the control group showed apparent renal tubular damage, renal tubular epithelial cell atrophy, degeneration, necrosis, and extensive lesion range. In the BA treatment group, the renal tubular epithelial injury was significantly improved compared with the control group, the lesion was lighter, and the lesion range was smaller (Figure 1D). BA treatment significantly reduced the tubular injury Paller score in IRI mice (Figure 1E). All of the aforementioned indicate that BA has a significant effect in alleviating the decline of renal function and tubular damage caused by IRI.

### Butyric Acid Regulates Foxp3 Expression, Cell Expansion, and the Function of CD4^+^ iTregs and Inhibited IL-17A^+^ Treg Cell Differentiation *in vitro*

**FIGURE 2:**
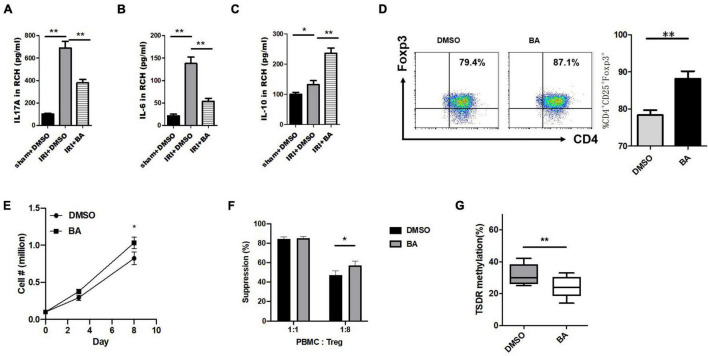
BA regulates Foxp3 expression, cell expansion, and the function of CD4^+^ iTregs and inhibited IL-17A^+^ Treg cell differentiation *in vitro*. **(A–C)** Renal cortex homogenate was collected to assay the anti-inflammatory effects of BA. The inflammatory cytokines IL-17A, IL-6 were significantly decreased after the treatment of BA in kidney IRI, while IL-10 was significantly increased. CD4^+^ cells (CD4^+^CD62L^High^CD44^Low^) isolated from C57/B6J mouse spleens were cultured under Treg polarizing conditions with BA or placebo. The expression of Foxp3 was significantly increased in the cells treated by BA within 8 days after induction. Compared with the control group, the absolute number of CD4^+^ iTregs in BA group was slightly increased on day 8 while the difference was not significant on day 8. The suppressive ability of BA-CD4^+^ iTregs was estimated by CFSE coculture assays. Although there was no difference in suppressive ability at 1:1 (Treg:CD4^+^ effector T cells), BA-CD4^+^ iTregs showed stronger inhibition at 1:8. We used bisulfite sequence analysis to detect the methylation status of CD4^+^ iTregs induced by BA. And compared with the control group, there were fewer methylated CpG sites in BA-CD4^+^ iTregs. Sample size = 3 in each group. Data were presented as mean ± standard deviation. N.S. *P* > 0.05, *P < 0.05, **P < 0.01.

Next, renal cortex homogenate was collected to assay the anti-inflammatory effects of BA. The inflammatory cytokines IL-17A and IL-6 (Proinflammatory factor) were significantly decreased after the treatment of BA in kidney IRI while IL-10 (anti-inflammatory factor) was significantly increased (Figures 2A–C). Treg was involved in the process of liver and kidney IRI (Zheng et al., 2018; Luan et al., 2020). Thus, we designed a series of *in vitro* experiments to test the effect of BA on the regulation of iTreg generation. Human naïve CD4^+^CD25^–^CD45RA^+^ T cells (purity ≥ 95%) were extracted from peripheral blood samples provided by healthy donors and then induced naïve CD4^+^ T cells to iTregs by TGF-β, IL-2, and CD3/CD28 beads.

Next, we explored at the cellular level whether BA regulated Treg differentiation *in vitro*. For this reason, CD4^+^ cells (CD4^+^CD62L^High^CD44^Low^) isolated from C57/B6J mouse spleens were cultured under Treg polarizing conditions with BA or placebo.

The expression of FoxP3 was significantly increased in the cells treated by BA after induction (Figure 2D). In addition, compared with the control group, the absolute number of CD4^+^ iTregs in the BA group was slightly increased on day 8 while the difference was not significant on day 8 (Figure 2E). The suppressive ability of BA-CD4^+^ iTregs was estimated by carboxyfluorescein succinimidyl amino ester (CFSE) coculture assays. The washed CD4^+^ iTregs were cocultured with CFSE-labeled fresh PBMCs at different ratios (Tregs: PBMCs = 1:1 or 1:8) in the presence of expansion beads. Figure 2F shows the expansion of CD4^+^ effector T cells. Although there was no difference in suppressive ability at 1:1 (Treg:CD4^+^ effector T cells), BA-CD4^+^ iTregs showed stronger inhibition at 1:8. Studies have confirmed that there is a significant correlation between pro-inflammatory/anti-inflammatory cytokines and Treg function (Pandiyan and Zhu, 2015; Qiu et al., 2018). These results suggest that BA inhibits the differentiation of naïve CD4^+^ T cells into other inflammatory cells and partly increases the function of CD4^+^ iTregs by regulating the expression of cytokines. The demethylation of CpG islands caused by epigenetic regulation of the Foxp3 loci is considered to be an important marker of the stability and functionality of Tregs (Floess et al., 2007). We used bisulfite sequence analysis to detect the methylation status of CD4^+^ iTregs induced by BA, and compared with the control group, there were fewer methylated CpG sites in BA-CD4^+^ iTregs (Figure 2G). Thus, BA regulates Treg cell differentiation and keeps its regulatory phenotype *in vitro*.

### Butyric Acid*BA* Regulates Treg/Th17 Balance by Targeting SOCS3 but Not SOCS1 in Renal Ischemia–Reperfusion Injury

**FIGURE 3:**
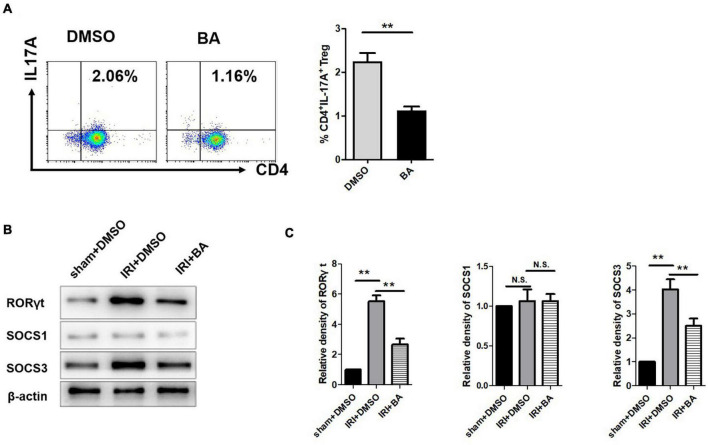
BA regulates Treg/Th17 balance by targeting SOCS3 but not SOCS1 in renal IRI. **(A)** The frequency of Th17 cells in the BA treatment group was significantly reduced. **(B,C)** The expression of RORγt and SOCS3, but not SOCS1 was decreased after BA treatment. Sample size = 3 in each group. Data were presented as mean ± standard deviation. N.S. *P* > 0.05, *P < 0.05, **P < 0.01.

Treg and Th17 cells are critical immune cells and play an essential role in various inflammatory diseases including renal IRI (Dellepiane et al., 2020). Flow cytometry was then used to detect the percentage of Th17 cells in mice blood. The addition of BA significantly reduced the frequency and number of CD4^+^IL-17A^+^ cells, promoting the inflammatory responses in renal IRI (Figure 3A). Our study found that the frequency of Th17 cells in the BA treatment group was significantly reduced, suggesting that BA regulates Treg/Th17 balance and regulates CD4^+^ T cell differentiation. Suppressor of cytokine signaling family (SOCS1/SOCS3) is necessary for the balance of Treg/Th17 population in different organ diseases (Qu et al., 2017; Wang D. et al., 2018; Kmiolek et al., 2020; Saleh et al., 2020). Therefore, we detected the expression of RORγt, SOCS1, and SOCS3 expression. As indicated in Figures 3B,C, the expression of RORγt and SOCS3, but not SOCS1, was decreased after BA treatment. These results suggest that BA regulates Treg/Th17 balance by targeting SOCS3 but not SOCS1 in renal IRI.

### Butyric Acid Protects Against Renal Ischemia–Reperfusion Injury *via* p-STAT3

**FIGURE 4:**
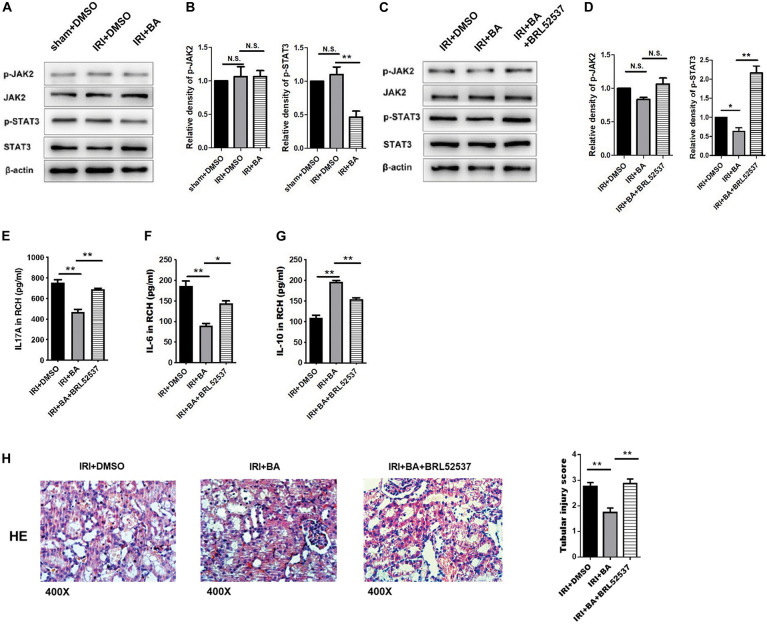
BA protects against renal IRI *via* p-STAT3/SOCS3 signaling. **(A,B)** BA treatments attenuated the expression of p-STAT3 but not p-JAK2. **(C,D)** After BRL52537 treatment, the expression of p-STAT3 but not p-JAK2 was increased. **(E–G)** BRL52537 significantly attenuated the anti-inflammatory effects of BA on renal IRI. **(H)** HE staining indicated that BRL52537 significantly attenuated the protective effects of BA on renal IRI. Sample size = 3 in each group. Data were presented as mean ± standard deviation. N.S. *P* > 0.05, *P < 0.05, **P < 0.01.

Foxp3 and RORγt mRNA and JAK2, STAT3, and SOCS3 protein are essential for the balance of Treg/Th17. We further tested the expression of p-JAK2, JAK2, p-STAT3, and STAT3 by western blot. As shown in Figures 4A,B, BA treatments attenuated the expression of p-STAT3 but not p-JAK2.

Since BRL52537, a kappa-opioid receptor agonist, could regulate p-STAT3 but not STAT3 expression in cerebral IRI and is involved in kidney injury, we used BRL52537 to assess whether p-STAT3 is necessary for the protective effects of BA (Fang et al., 2013; Golosova et al., 2020). After BRL52537 treatment, the expression of p-STAT3 but not p-JAK2 was increased, which conformed to the p-STAT3 agonist role of BRL52537 (Figures 4C,D). The previous studies show that overexpression of STAT-3 would increase the percentages of Th17 and relevantly reduce the rates of Treg cells *in vivo* and *in vitro* (Wang Y. et al., 2018; Liu et al., 2020). We observed similar results after BRL52537 treatment in our results (data not shown). Renal cortex homogenate was collected to assay the inflammatory effects after the increased expression of p-STAT3. Figures 4E–G suggests that BRL52537 significantly attenuated the anti-inflammatory effects of BA on renal IRI. Also, HE staining indicated that BRL52537 significantly attenuated the protective effects of BA on renal IRI (Figure 4H). Taken together, BA exerts therapeutic and protective effects on renal IRI through p-STAT3.

### HO-1/p-STAT3 Signaling Pathway Was Implicated in Treg/Th17 Balance Mediated by Butyric Acid

**FIGURE 5:**
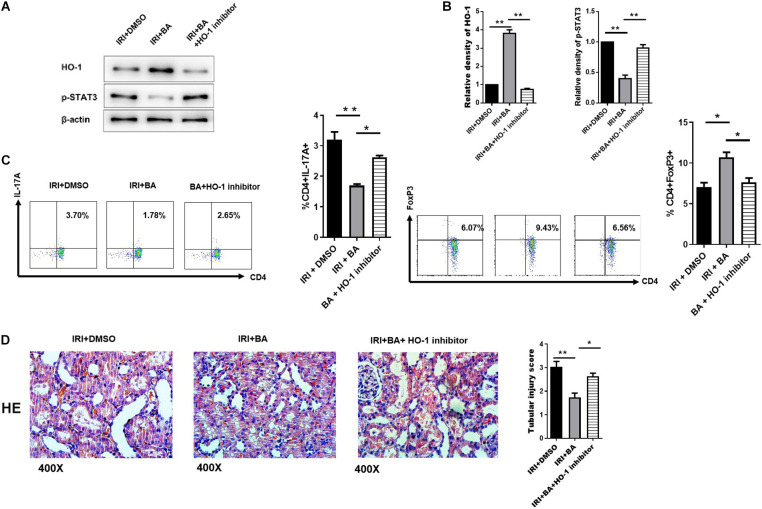
HO-1/p-STAT3 signaling pathway was implicated in Treg/Th17 balance mediated by BA. **(A,B)** SnPP (the inhibitor of HO-1), significantly reduced the up-regulation of BA-related p-STAT3 expression. **(C)** In CD4^+^ T cells under Treg/TH17 polarizing conditions. BA/Snpp co-treatment reversed the immunological balance of Treg/Th17 cells by BA only. **(D)** HE staining show that SnPP treatment significantly, but not wholly, reversed the regulating effect of BA. Sample size = 3 in each group. Data were presented as mean ± standard deviation. N.S. *P* > 0.05, *P < 0.05, **P < 0.01.

Butyric acid is an effective activator of Nrf2, while Nrf2/HO-1 signaling pathway is an essential regulator of oxidative stress. The researches show that HO-1 mediates STAT3 pathway in different cells and diseases (Tang et al., 2018; Kuang et al., 2019). Therefore, we decided to identify whether the signaling factor HO-1 was implicated in Treg/Th17 cell differentiation mediated by BA. As shown in Figures 5A,B, SnPP, the inhibitor of HO-1, significantly reduced the upregulation of BA-related p-STAT3 expression. In CD4^+^ T cells under Treg/TH17 polarizing conditions, BA/SnPP co-treatment reversed the immunological balance of Treg/Th17 cells by BA only (Figure 5C). Also, the pathological results show that SnPP treatment significantly, but not wholly, reversed the regulating effect of BA (Figure 5D). To conclude, HO-1/p-STAT3 signaling pathway was implicated in Treg/Th17 balance mediated by BA in renal IRI.

## Discussion

Short-chain fatty acids, such as acetic acid, propionic acid, and BA, are produced by gut microbes during the fermentation of dietary fibers. BA, a critical SCFA, as a significant energy source for enterocytes, has many physiological functions, including maintaining the balance of intestinal flora and regulating electrolytes and the body-fluid balance. More importantly, BA can inhibit the release of inflammatory cytokines, preserve the integrity of the intestinal mucosal barrier, and improve the function of the intestinal immune system (Hofmanova et al., 2014; Louis et al., 2014). As a histone deacetylase inhibitor (HDACi) (Tan et al., 2014; Cleophas et al., 2016), BA plays a vital role in gene transcription. BA can increase the expression of Fas protein in T cells by inhibiting the acetylation of histones, thus inducing the apoptosis of T cells and inhibiting the inflammatory response (Zimmerman et al., 2012). When inflammatory response occurs, inflammatory cells produced free lipopolysaccharide (LPS), and inflammatory cytokines as well as chaperone protein STUB1. At the same time, FoxP3 degrades after ubiquitination in the presence of chaperone HSP70 (Chen et al., 2013; Laurence et al., 2013). Studies have shown that BA indirectly inhibited the degradation of Foxp3 by inhibiting IL-6 expression (Vinolo et al., 2011). Furusawa et al. (2013), Hoeppli et al. (2015) demonstrated that BA could also act with HDACi on both initial T cells and Treg cells, increasing the acetylation of histone H3 in the conservative non-coding sequence of FoxP3 (CNS-1), which promotes the differentiation of initial T cells into Treg cells on the one hand, and increases the expression of Foxp3 protein in Treg cells on the other hand. Besides, Vinolo et al. (2011), Smith et al. (2013) demonstrated that drinking water containing BA in sterile mice increased the number of Treg cells in the intestinal mucosa lamina propria of mice and promoted Treg cell synthesizing the inflammatory suppressor IL-10. Therefore, BA can promote the differentiation of Treg cells and the expression of FoxP3 and IL-10, thus regulating the inflammatory response.

Th17 and Treg cells, which belong to CD4^+^ T cells, have gained much attention. The initial CD4^+^ T cells differentiated into Treg cells induced by TGF-β and differentiated into Th17 cells under the combined action of TGF-β and IL-6 or IL-21. Th17 cells produce IL-17, IL-22, and IL-23, recruited neutrophils, and promoted inflammation at the injection site. On the contrary, after being activated by homologous antigens, Treg cells play a negative immunomodulatory role, mainly secreting anti-inflammatory cytokines such as IL-10 and TGF-β to inhibit the activities of various immune cells, thus suppressing the immune response (Hofmanova et al., 2014). According to many rodent studies, BA increases Tregs and reduces the differentiation of other Th cells (Cao et al., 2018; Luu et al., 2018). As demonstrated by Hu et al. (2018), the development of autoimmune hepatitis can be attenuated through regulation of the imbalance of Treg/Th17 and intestinal barrier function by a high-fiber diet. In addition, inflammatory skin reactions can be mitigated by SCFA sodium BA through inducing Tregs (Schwarz et al., 2017). However, BA also regulates the expression of several cytokines secreted by CD4^+^ Tregs, which increases the suppressive ability and decreases the instability of Tregs. The mechanism underlying these effects will be investigated further and considered for future clinical applications.

Vinolo et al. (2011), Tan et al. (2014) suggested that BA can activate GPRs, especially GPR43 and GPR109a; reduced the synthesis and secretion of pro-inflammatory factors, reactive oxygen species (ROS), and cyclooxygenase (COX2); and inhibited inflammatory responses. Sun and Ye (2012)) demonstrated that BA could affect its downstream MEK-ERK pathway *via* activating GPRs, thereby indirectly contributing to the secretion of antimicrobial peptide LL37, ultimately suppressing the inflammatory response. Klampfer et al. (2003) identified that BA inhibited the activity of JAK2 and suppressed IFNγ-induced tyrosine and serine phosphorylation of STAT1, thereby inhibiting the activity of the JAK-STAT pathway and ultimately reducing proinflammatory cytokine synthesis. Many experimental studies have shown that BA can inhibit the release of inflammation-related factors by inhibiting intracellular signaling pathways.

HO-1 is a key star molecule in the process of oxidative stress, and the role of HO-1 in inflammatory response has also been widely recognized (Sun et al., 2021; Wang et al., 2021; Zhang et al., 2021). In recent years, the relationship between HO-1 and immunity has gained some attention (Yan et al., 2019; Van Nguyen et al., 2020). However, whether HO-1 plays an essential role in the immune response to renal IRI remains unclear. Our research shows that HO-1 mediates the protection of BA against renal IRI, and this protective effect is related to the balance of Treg/Th17. We believe that HO-1 mediates the regulation of the Treg/Th17 balance. Similar to our research conclusion, the research of Zhang et al. (2018) showed that HO-1 agonist can increase Tregs in patients with vitiligo. They found that HO-1 restored the function of Tregs by upregulating the expression of IL-10, proved that HO-1 could significantly promote Treg expression in patients with vitiligo, and showed the potential of HO-1 as a therapeutic target for vitiligo Zhang et al. (2018). Another study showed that HO-1 might show anti-inflammatory activity in the mouse model of acute experimental colitis by regulating the balance between Th17 and Treg cells, thus providing a new therapeutic target for inflammatory bowel disease (Zhang et al., 2014). Treg/Th17 balance is important for multi-factor regulation. The JAK2-STAT3 signaling pathway is a classic signaling pathway that regulates the differentiation of CD4^+^ T cells. Influencing the activation of the JAK2-STAT3 signaling pathway can regulate the differentiation of CD4^+^ T cells, thereby regulating the balance of Treg/Th17. Our research shows that under the intervention of BA, HO-1 significantly affects the activation of the JAK2-STAT3 signaling pathway. HO-1 regulates p-STAT3 to affect the immune-inflammatory response during renal IRI. The research of Lin et al. (2017) corroborates our findings. Their research shows that HO-1 directly binds to STAT3 to control the production of pathogenic Th17 cells during neutrophil airway inflammation. They also creatively clarified HO-1 regulates the site of p-STAT3 (Lin et al., 2017). HO-1 is not only a key molecule of oxidative stress but also participates in the immune response process of a variety of diseases by regulating p-STAT3 (Chiang et al., 2020; Levin-Epstein et al., 2020; Lin et al., 2020). There is a certain link between oxidative stress and immune-inflammatory response that cannot be ignored.

Many studies have proved that Treg cells play an essential role in inflammatory bowel disease, transplantation immunity, bronchial asthma, and other diseases (Gibson et al., 2013; Singer et al., 2014; Terhune and Deth, 2014). The most prominent feature of Treg cells is the expression of Foxp3 and the demethylation of the Foxp3 gene locus (Hoeppli et al., 2015). In recent years, experiments have confirmed that by regulating Th17/Treg cell balance, the severity of renal IRI could be alleviated (Gan et al., 2019). Cleophas et al. (2016) also found that in the renal IRI model, IL-2C could regulate Th17/Treg cell balance by increasing Treg expression, thus reducing renal IRI. In the current study, we demonstrated that BA protected against renal IRI by enhancing CD4^+^ Tregs and keeping the balance of the Treg/Th17 population. *In vitro* experiments showed that BA improved human Treg generation following IL-2 and TGF-β stimulation *via* promoting Foxp3 expression and suppressing T cell expansion.

## Conclusion

In conclusion, the present study demonstrated that BA directly decreased Th17 cells and increased Treg cells, thus reducing the inflammatory response. Importantly, we further identified that BA protected against renal IRI *via* HO-1/p-STAT3 signaling, which was implicated in Th17 cell differentiation. Our results provided ideas that BA can inhibit renal IRI by regulating Treg/TH17 cell balance and being a potential therapeutic option for renal IRI after surgery.

## Data Availability Statement

The original contributions presented in the study are included in the article/supplementary material, further inquiries can be directed to the corresponding authors.

## Ethics Statement

The studies involving human participants were reviewed and approved by the Ethics Committee of the Third Affiliated Hospital of Soochow University. The patients/participants provided their written informed consent to participate in this study. The animal study was reviewed and approved by the Institutional Animal Care and Use Committee of Soochow University.

## Author Contributions

ZC and YJL: Conception and design. MMW, SKY, JS, THJ, and WD: Analysis and interpretation. MMW, SKY, JS, THJ, and WD: Data collection. ZC, YJL, and LHJ: Writing the manuscript. ZWF and JC: Critical revision of the manuscript. ZWF, JC, and YJL: Final approval of the manuscript. All authors contributed to the article and approved the submitted version.

## Conflict of Interest

The authors declare that the research was conducted in the absence of any commercial or financial relationships that could be construed as a potential conflict of interest.

## Publisher’s Note

All claims expressed in this article are solely those of the authors and do not necessarily represent those of their affiliated organizations, or those of the publisher, the editors and the reviewers. Any product that may be evaluated in this article, or claim that may be made by its manufacturer, is not guaranteed or endorsed by the publisher.
