# Corrigendum: Butyric acid protects against renal ischemia–reperfusion injury by adjusting the Treg/Th17 balance *via* HO-1/p-STAT3 signaling

**DOI:** 10.3389/fcell.2022.999965

**Published:** 2022-08-24

**Authors:** Zhen Chen, Miaomiao Wang, Shikun Yang, Jian Shi, Tianhao Ji, Wei Ding, Lianghua Jiang, Zhiwen Fan, Jing Chen, Yunjie Lu

**Affiliations:** ^1^ The Third Affiliated Hospital of Soochow University, Changzhou, China; ^2^ Key Laboratory of Liver Transplantation, Hepatobiliary/Liver Transplantation Center, The First Affiliated Hospital of Nanjing Medical University, Chinese Academy of Medical Sciences, Nanjing, China; ^3^ Wujin Hospital Affiliated With Jiangsu University, Changzhou, China; ^4^ The First People’s Hospital of Kunshan, Kunshan, China; ^5^ Department of Pathology, Affiliated Nanjing Drum Tower Hospital of Nanjing University School of Medicine, Nanjing, China

**Keywords:** butyric acid, renal ischemia–reperfusion injury, Treg, Th17, HO-1, STAT3

In the published article, there was an error in Figure 4H as published. We applied the incorrect picture to Figure 4H. The corrected Figure 4H and its caption “IRI + BA + BRL52537” appear below.

**FIGURE 4:**
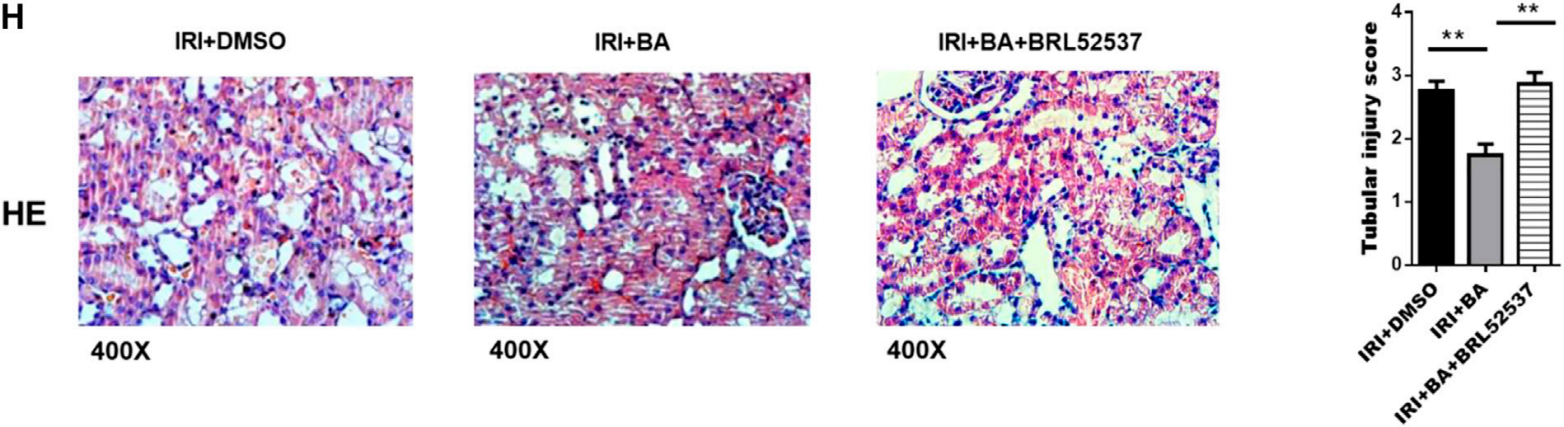
BA protects against renal IRI *via* p-STAT3/SOCS3 signaling. **(A,B)** BA treatments attenuated the expression of p-STAT3 but not p-JAK2. **(C,D)** After BRL52537 treatment, the expression of p-STAT3 but not p-JAK2 was increased. **(E–G)** BRL52537 significantly attenuated the anti-inflammatory effects of BA on renal IRI. **(H)** HE staining indicated that BRL52537 significantly attenuated the protective effects of BA on renal IRI. Sample size = 3 in each group. Data were presented as mean ± standard deviation. N.S. *p* > 0.05, *p < 0.05, **p < 0.01.

The authors apologize for this error and state that this does not change the scientific conclusions of the article in any way. The original article has been updated.

